# Prevalence of Self-Reported Shaking and Smothering and Their Associations with Co-Sleeping among 4-Month-Old Infants in Japan

**DOI:** 10.3390/ijerph110606485

**Published:** 2014-06-20

**Authors:** Fujiko Yamada, Takeo Fujiwara

**Affiliations:** 1 Department of Social Medicine, National Research Institute for Child Health and Development, Setagaya, Tokyo 157-8535, Japan; E-Mail: y-fujiko@mue.biglobe.ne.jp; 2 Department of Developmental Social Medicine, Mie University Graduate School of Medicine, Tsu, Mie 514-8507, Japan

**Keywords:** abusive head trauma, shaken baby syndrome, shaking, smothering, child abuse, co-sleeping, crying, Japan

## Abstract

Few studies have investigated the prevalence of shaking and smothering and whether they are associated with co-sleeping. In Japan, co-sleeping is common during infancy and early childhood. This study investigates the prevalence of shaking and smothering and their associations with co-sleeping among 4-month-old infants in Japan. A questionnaire was administered to mothers who participated in a 4-month health checkup program in Kamagaya City in Japan (n = 1307; valid response rate, 82%). The questionnaire investigated the frequency of self-reported shaking and smothering during the past one month, co-sleeping status, and living arrangements with grandparents, in addition to traditional risk factors such as stress due to crying. Associations between co-sleeping and self-reported shaking or smothering were analyzed using multiple logistic regression. The prevalence of self-reported shaking and smothering at least one time during the past one month was 3.4% (95% confidence interval (CI), 2.4%–4.3%) and 2.4% (95% CI, 1.5%–3.2%), respectively. Co-sleeping was marginally associated with the amount of crying and not associated with stress due to crying. Further, co-sleeping was not associated with either self-reported shaking or smothering, although stress due to crying showed strong association with shaking and smothering. Co-sleeping was not a risk factor for shaking and smothering.

## 1. Introduction

Shaken baby syndrome (SBS) or abusive head trauma (AHT) is the leading cause of death due to child abuse [1]. To prevent SBS/AHT, it is important to determine their prevalence and risk factors to develop an efficient intervention program and evaluate its effectiveness. The well-known risk factor for shaking is frustration due to crying. In a Dutch study, 1.3% and 3.4% of parents of 3- and 6-month-old infants, respectively, had reported shaking their infants at least once, and worries about crying showed strong association with shaking [2]. However, these findings need to be replicated in a different parenting environment such as that in Japan, where the home environment is relatively small and co-sleeping is common [3,4]. In a co-sleeping environment, the distance between caregiver and infant is close. This might induce frustration when the infant cries a lot while the caregiver is sleeping. However, it may have beneficial effects such as reducing both infant crying (caregivers can respond earlier to infant distress) and caregiver stress (parents can respond directly to calm the infant instead of getting out of bed to attend to the infant). 

Similarly, smothering is also known as a form of life-threatening child abuse [5,6] that is associated with infant crying [2]. However, prevalence of smothering in Japan has not yet been reported, and the association between co-sleeping and smothering is unknown. Previous studies have shown that co-sleeping is regarded as a risk factor for unexpected death due to suffocation [7] or sudden infant death syndrome [8]. Although a small number of cases of smothering when co-sleeping might be due to intentional smothering, most cases are due to non-intentional smothering [9]. Thus, the purpose of this study was to investigate the prevalence of shaking and intentional smothering and their associations with co-sleeping among 4-month-old infants in Japan.

## 2. Method

### 2.1. Sample

The subjects were all mothers (n = 1594) who participated in a 4-month health checkup program between June 2010 and January 2012 in Kamagaya City in the Chiba Prefecture, located next to Tokyo, Japan. Kamagaya City is located in a suburban area northwest of Chiba City, Chiba Prefecture, with a population of approximately 108,000 and approximately 1000 births per year. An anonymous questionnaire was mailed directly to eligible mothers before the start of the 4-month health checkup program. Responses were collected during each health checkup. In total, 1334 mothers responded (response rate, 84%). Our study was approved by the ethics committee of the National Institute for Public Health, which determined that it was no longer necessary to obtain consent from participants, given that responses to the questionnaire already implied consent to participate in the study. We informed participants of the nature and purpose of this study and the safeguards in place to protect anonymity in the questionnaire. We also explained at the health check up to ensure that participants understood that they had a genuine choice to participate or not, which was explained at the time of health checkup.

#### 2.1.1. Shaking and Smothering Measures

Shaking the child when crying was assessed as self-reported shaking by the 4-month questionnaire. Frequency of self-reported shaking in the last month was recorded using the following response items: “0 times”, “1 or 2 times”, “3–5 times”, “6–10 times”, and “11 or more times”. Because the term “shaking” may be misunderstood as “rocking” in Japanese, in the questionnaire we used the Japanese term for “violently shaking” instead. Self-reported smothering was assessed using the following question: “How many times have you ever smothered the mouth of your baby when crying, using your hands, a cushion, *etc.* during the last month?” The same response items as those for shaking were used.

#### 2.1.2. Co-sleeping and Other Covariates

Co-sleeping was assessed in the 4-month questionnaire by the following question: “Do you share your futon or bed with your infant when you sleep?”, followed by the question, “Do you sleep with your infant in the same room?”, to differentiate between room-sharing and bed/futon sharing (*i.e.*, co-sleeping). Other possible covariates include marital status, living together with grandparents, housing type, annual household income, maternal education, infant age, sex, being firstborn, birth weight, and feeding type. Further, stress due to infant crying during the last month and the amount of crying were also assessed. Stress due to infant crying was assessed based on the mother’s rating of the questionnaire item “feel stress due to crying during the last month” using a 5-point Likert scale, with 1 indicating “Not at all” and 5 indicating “Always”. We then defined low stress as “Not at all”, middle stress as “A little” or “Sometimes”, and high stress as “Often” or “Always”. The amount of crying was assessed based on the response to “my baby was crying a lot” using the 5-point Likert scale. We then defined a small amount of crying as “Not at all” or “Rather no”, a medium amount of crying as “Neither”, and a large amount of crying as “Yes, a lot” or “Rather yes”. 

### 2.2. Analysis

We analyzed the data with valid responses on co-sleeping, shaking, smothering, amount of crying, and stress due to crying (n = 1307). First, the prevalence of shaking and smothering was calculated with a 95% confidence interval (CI). Second, the association of co-sleeping with amount of crying and stress due to crying was assessed by chi-square test. Third, associations between co-sleeping and self-reported shaking or smothering were dichotomized based on frequency as 0 times (no) or >1 times (yes), and were analyzed using multiple logistic regression adjusted for covariates. All analyses were conducted using Stata/MP v12.0 software (StataCorp LP, College Station, TX, USA).

## 3. Results

Participants’ characteristics are presented in Table 1. Almost all participants were married (98.3%), and most couples were considered to be living together according to a nationally representative sample [10]. Of all participants, 10.6% were living with grandparents. Household income ranged from an annual income of less than 2 million yen (2.6%), which was considered as severe poverty, to an annual income of 8 million yen or more (7.0%), which was considered affluent. Around half of the infants were firstborns who lived in detached houses, and were exclusively breastfed. Mothers who co-slept with their infants made up 51.7% of participants, which is more likely found if the infant was subsequent infant or exclusively breast-fed (both *p* < 0.001).

Further, the overall prevalence of shaking at least once during the last month was 3.4% (95% CI, 2.4%–4.3%). Similarly, the overall prevalence of smothering at least once during the last month was 2.4% (95% CI, 1.5%–3.2%), which was positively associated with living with grandparents (*p* = 0.006).

The association of co-sleeping with the amount of crying and stress due to crying is shown in Table 2. Most women reported a small amount of crying from infants (54.6%), and 20.7% reported a large amount. We observed a trend towards greater amounts of crying among infants who were co-sleeping, but this did not reach statistical significance. The majority of mothers reported middle stress due to infant crying (65.4%), suggesting that middle stress is the norm. Middle stress was not associated with co-sleeping status (*p* = 0.53).

The odds ratios (OR) of co-sleeping for shaking and smothering at least once during the past month are shown in Table 3. Co-sleeping was not associated with shaking (OR: 1.10, 95% CI: 0.54–2.26) and smothering (OR: 0.86, 95% CI: 0.38–1.94) in the adjusted model. On the contrary, mothers who experienced high stress due to infant crying showed strong association with shaking (OR: 3.10, 95% CI: 1.28–7.48) and smothering (OR: 3.54, 95% CI: 1.27–9.88) in comparison with mothers who experienced middle stress.

## 4. Discussion

The prevalence of self-reported shaking and smothering among 4-month-old infants in Japan was 3.4% and 2.4%, respectively, indicating that these abusive behaviors are not rare. To the best of our knowledge, the present study is the first to report on the prevalence of self-reported shaking and smothering in Japan, where houses are relatively small and co-sleeping is common [3,4]. The prevalence of self-reported shaking in our study is similar to those of previous studies in The Netherlands (1.3% and 3.4% of parents of 3- and 6-month-old infants) [2] and the United States (2.6% of parents of <2-year-old children) [11]. Moreover, we observed a self-reported smothering prevalence in Japan (2.4% among 4-month-old infants) similar to that in The Netherlands (1.3% and 1.6% among 3- and 6-month-old infants, respectively) [2]. It is interesting that our prevalence figures are similar to those previously reported in Western countries.

Our results suggest no substantial association between co-sleeping and the amount of infant crying or stress caused by crying. That is, close proximity of the parent to the infant may not influence the amount of crying, at least at the age of 4 months, which is consistent with the literature [12]. Moreover, it is noteworthy to mention that sleeping separately in the same room (*i.e.*, no co-sleeping) may not be effective in decreasing stress due to crying.

**Table 1:** Characteristics of sample.

Characteristics		Total (n = 1307)		Co-sleeping (+) (n = 687, 51.7%)			Self-reported shaking (+) (n = 44, 3.4%)			Self-reported smothering (+) (n = 31, 2.4%)		
		n	%	n	%	*p*	n	%	*p*	n	%	*p*
Marital status	Married	1284	98.5	671	98.4	0.81	43	97.7	0.69	30	96.8	0.44
	Never married or divorced	20	1.5	11	1.6		1	2.3		1	3.2	
Living together with grandparents	No	1168	89.4	606	88.6	0.35	40	90.9	0.74	23	74.2	**0.006**
	Yes	139	10.6	78	11.4		4	9.1		8	25.8	
Housing type	Apartment	650	50.2	323	47.7	0.066	28	63.6	0.069	13	43.3	0.45
	Detached house	646	49.9	354	52.3		16	36.4		17	56.7	
Annual household income (million yen)	<2	34	2.6	21	3.1	0.15	3	6.8	0.25	2	6.5	0.47
	2.1–4	383	29.3	215	31.4		14	31.8		8	25.8	
	4.1–6	445	34.1	234	34.2		12	27.3		14	45.2	
	6.1–8	204	15.6	100	14.6		5	11.4		3	9.7	
	>8	92	7.0	48	7.0		2	4.6		1	3.2	
	No answer	149	11.4	66	9.7		8	18.2		3	9.7	
Infant sex	Boy	662	50.8	348	51.1	0.82	21	47.7	0.68	18	58.1	0.41
	Girl	641	49.2	333	48.9		23	52.3		13	41.9	
First baby	Yes	642	49.2	289	42.4	**<0.001**	19	43.2	0.31	10	32.3	**0.037**
	No	662	50.8	393	57.6		25	56.8		21	67.7	
Feeding type	Breastfeeding only	618	47.4	380	55.6	**<0.001**	14	31.8	0.101	15	48.4	0.76
	Mixed	410	31.4	195	28.6		17	38.6		11	35.5	
	Bottle only	276	21.2	108	15.8		13	29.6		5	16.1	

**Table 2:** Association of co-sleeping with amount of crying, and stress due to crying.

Characteristics on Crying		Total		Co-sleeping (+)		
		n	%	n	%	*p*
Amount of crying	Small	713	54.6	363	53.1	0.072
	Medium	324	24.8	163	23.8	
	Large	270	20.7	158	23.1	
Stress due to infant crying	Low	384	29.4	206	30.1	0.53
	Middle	855	65.4	439	64.2	
	High	68	5.2	39	5.7	

**Table 3:** Odds ratio of co-sleeping and crying variables for self-reported shaking and smothering.

Variables		Self-reported Shaking								Self-reported Smothering			
		Crude				Adjusted *				Crude		Adjusted *	
		n	%	OR	95% CI	OR	95% CI	n	%	OR	95% CI	OR	95% CI
Co-sleeping	Yes	24	3.5	1.10	0.60–2.00	1.10	0.54–2.26	17	2.5	1.11	0.54–2.27	0.86	0.38–1.94
	No	20	3.2	ref		ref		14	2.3	ref		ref	
Amount of crying	Small	11	25.0	ref		ref		12	38.7	ref		ref	
	Medium	10	22.7	2.03	0.85–0.83	2.26	0.86–5.96	5	16.1	0.92	0.32-2.62	0.71	0.23–2.14
	Large	23	52.3	**5.94**	**2.86–12.37**	**3.68**	**1.47-9.20**	14	45.2	**3.19**	**1.46–7.00**	1.50	0.58–3.86
Stress due to infant crying	Low	2	4.6	**0.14**	**0.03–0.58**	0.25	0.06–1.09	2	6.5	**0.20**	**0.05–0.85**	0.23	0.05–1.05
	Middle	31	70.5	ref		ref		22	71.0	ref		ref	
	High	11	25.0	**5.13**	**2.45–10.73**	**3.10**	**1.28–7.48**	7	22.6	**4.35**	**1.79–10.57**	**3.54**	**1.27**–**9.88**

Note: * Adjusted for amount of crying, stress due to infant crying, marital status, living with grandparents, housing type, income, child’s sex, first child, and feeding type. ref: reference category.

We found that co-sleeping was not associated with either shaking or smothering. This is important because we cannot hypothesize that close proximity during sleep may increase parental stress and therefore did not increase the risk of shaking and smothering. The alternative hypothesis that close proximity during sleep may decrease parental stress due to quick parental response to crying cannot be made either. Thus, co-sleeping cannot be presumed to be either a risk or protective factor for shaking or smothering.

On the contrary, we confirmed that stress due to infant crying is associated with self-reported shaking and smothering in our study, which is consistent with the findings of a previous study [13,14]. In the Dutch study, parents who were worried about their child crying sometimes or frequently were 3.05 times more likely to shake, smother, or slap their infant than those who never worried about their child crying [2]. Our study investigated stress due to infant crying and obtained similar ORs with shaking (high *vs.* middle stress level, 3.10) and smothering (high *vs.* middle stress level, 3.54). Further, we add to the literature that dose-response associations with stress due to crying and both shaking and smothering were observed. 

We found that living with grandparents, a proxy measure for family density, was positively associated with smothering, which highlights the importance of educating cohabitants, such as grandparents, on the characteristics of crying. However, this association might be specific to Japan, where caregivers tend to be anxious that the infant’s crying might bother cohabitants (e.g., grandparents) or neighbors [15]. Thus, educating mothers and caregivers on how to manage a crying infant is needed in order to develop a preventive strategy against smothering.

Several limitations of the present study need to be addressed. First, shaking and smothering were self-reported, not based on objective measurements such as video recordings or diary records, although a previous study used a self-administered questionnaire to assess the prevalence of shaking and smothering [2,16]. Moreover, it is difficult to know how these self-reported cases link to infants who are admitted to hospital for SBS/AHT. In addition, the majority of mothers who reported shaking or smothering their child on 1–2 times occasions only. It is not clear whether the parent and researchers interpreted the meaning of “smothering” or “violent shaking” in the same way. Second, the cases of self-reported shaking and smothering might have been misclassified, although we clearly defined “shaking” and “smothering” in the questionnaire. The interpretation of shaking might be different in other cultures [16]; for this reason, we defined “shaking” as “violent shaking while the infant is crying.” Third, we did not investigate shaking and smothering by fathers, who are the major perpetrators of shaking and smothering in the U.S. [17,18], although in Japan one study observed that the majority of perpetrators of SBS/AHT in their study population tended to be mothers [19]. Thus, the prevalence of self-reported shaking or smothering can be underestimated. Fourth, as we assessed the prevalence of shaking and smothering in a single city near Tokyo, which was not a representative sample of Japan, this limits the generalizability of our findings. Therefore, further study is warranted using larger representative sample populations in Japan to replicate the prevalence of and risk factors for shaking and smothering. Fifth, although we conducted a population-based survey in the present study, not all participants responded to the survey, which might have caused underestimation of the prevalence of self-reported shaking and smothering because these behaviors might be more prevalent among non-respondents. Further study using routine health checkups in public health practice is needed to investigate shaking and smothering behaviors among caregivers of 4-month-old infants.

## 5. Conclusions

In conclusion, cases of shaking and smothering in Japan are not rare, with prevalence rates consistent with those of Western countries. Co-sleeping, which is common in Japan, was not associated with self-reported shaking or smothering. As co-sleeping was not associated with stress due to crying, we can conclude that co-sleeping cannot be a risk factor for shaking or smothering. 
